# Multi‐scale relief model (MSRM): a new algorithm for the visualization of subtle topographic change of variable size in digital elevation models

**DOI:** 10.1002/esp.4317

**Published:** 2018-02-05

**Authors:** Hector A. Orengo, Cameron A. Petrie

**Affiliations:** ^1^ McDonald Institute of Archaeological Research University of Cambridge Cambridge UK; ^2^ Department of Archaeology and Anthropology University of Cambridge Cambridge UK

**Keywords:** digital terrain/surface model, feature detection and analysis, micro‐relief/topography visualization, palaeo‐hydrology/environment, Google Earth Engine

## Abstract

Morphological analysis of landforms has traditionally relied on the interpretation of imagery. Although imagery provides a natural view of an area of interest (AOI) images are largely hindered by the environmental conditions at the time of image acquisition, the quality of the image and, mainly, the lack of topographical information, which is an essential factor for a correct understanding of the AOI's geomorphology.

More recently digital surface models (DSMs) have been incorporated into the analytical toolbox of geomorphologists. These are usually high‐resolution models derived from digital photogrammetric processes or LiDAR data. However, these are restricted to relatively small areas and are expensive or complex to acquire, which limits widespread implementation.

In this paper, we present the multi‐scale relief model (MSRM), which is a new algorithm for the visual interpretation of landforms using DSMs. The significance of this new method lies in its capacity to extract landform morphology from both high‐ and low‐resolution DSMs independently of the shape or scale of the landform under study. This method thus provides important advantages compared to previous approaches as it: (1) allows the use of worldwide medium resolution models, such as SRTM, ASTER GDEM, ALOS, and TanDEM‐X; (2) offers an alternative to traditional photograph interpretation that does not rely on the quality of the imagery employed nor on the environmental conditions and time of its acquisition; and (3) can be easily implemented for large areas using traditional GIS/RS software.

The algorithm is tested in the Sutlej‐Yamuna interfluve, which is a very large low‐relief alluvial plain in northwest India where 10 000 km of palaeoriver channels have been mapped using MSRM. The code, written in Google Earth Engine's implementation of JavaScript, is provided as Supporting Information for its use in any other AOI without particular technical knowledge or access to topographical data. © 2017 The Authors. Earth Surface Processes and Landforms published by John Wiley & Sons Ltd.

## Introduction

Morphological analysis of landforms has traditionally relied on the interpretation of near‐vertical aerial and satellite images. Although imagery provides a natural plan‐view of an area of interest (AOI), effective interpretation is largely hindered by the environmental conditions at the time of image acquisition, the quality of the image and, most importantly, the lack of topographical information, which is an essential factor for a correct understanding of AOI morphology. Visual interpretation of aerial imagery has usually relied on assessment of the planimetric morphology of the features of interest and the image elements providing indications of elevation change, such as shadowed areas. Although aerial photography time series capacity to measure geomorphic change cannot be currently matched, the problems associated to the use of imagery for the interpretation of landforms have been long recognized (e.g. Shroder, 2013, 29–30; Liu and Coulthard, 2015, 247).

More recently digital topographies have been incorporated into the analytical toolbox of geomorphologists. Digital terrain models (DTMs) and digital surface models (DSMs) are types of digital elevation models (DEMs) that replicate terrain and surface topography. These models have become a common source of geographical analysis and are routinely employed in a large number of disciplines with a geographical basis, including engineering, geomorphology, hydrology, landscape architecture, and archaeology. With the wider availability of high‐resolution topographic data derived from photogrammetric models and LiDAR (e.g. Lane *et al*., 2003; Zamora, 2017), it has become evident that traditional methods of DEM visualization, such as colour scaling and shaded relief, are insufficient for reflecting small variations in height. These ‘micro‐topographies’ are, however, capable of revealing important geomorphological and cultural information. In order to increase the visibility of micro‐reliefs a series of DSM filtering techniques have been developed in recent years. Some techniques, like Sky View Factor (Zakšek *et al*., 2011), Openness (Yokoyama *et al*., 2002), I‐Factor (Lin *et al*., 2013) and principal component analysis (PCA) of multi‐azimuth shaded relief maps (Devereux *et al*., 2008), are based on digital illumination of the surface, while others, such as Slope Gradient (Doneus and Briese, 2006; Challis *et al*., 2011) and Local Relief Model (Hesse, 2010), are based on topographic filtering and analysis. Combinations of these methods have also been attempted (Mayoral *et al*., 2017). In general, these techniques were designed to highlight small‐scale micro‐topographies in high‐resolution datasets, particularly LiDAR‐derived DTMs. However, micro‐topographies do not necessarily need to be small in plan size. Features such as palaeorivers, levees and dunes – all of which are features of interest examined here – might only present a slight topographic variation with respect to their surrounding terrain, and may also extend over hundreds of metres. Although they are large in area, such subtle topographic variations would not be detected by any of the traditional methods, and as a result, large‐scale micro‐topographies have remained difficult to detect. A solution to this would be to increase the kernel of the filter employed for the specific filtering method employed, but the variability in the size of features of interest typically results in only partial detection, leaving the features that do not fit within the specified kernel size largely unidentified.

High‐resolution DSMs contain abundant information and are usually available for small areas and exceptionally at national level, but their use is still restricted due to their often prohibitive pricing if commercially acquired, as well as the specialized hardware and technical expertise required for their generation, and their limited spatial coverage. In contrast, medium resolution DSMs (2–30 m/cell), such as the 30 m/cell SRTM, ASTER GDEM and ALOS World 3D, are freely available and cover most of the Earth surface. Depending on the scale of the features being sought, such medium resolution elevation data has the potential to contain important multi‐scale topographic information that cannot be extracted using standard visualization techniques or current filtering methods. In this regard, the development of techniques able to fully exploit the still largely untapped topographic information in medium‐resolution DSMs for the detection of micro‐reliefs has enormous potential to contribute to geomorphological research and beyond.

This paper presents a new algorithm, multi‐scale relief model (MSRM), which is able to extract micro‐topographic information at a variety of scales employing micro‐, meso‐ and large‐scale DSM/DTMs. The algorithm was originally developed to complement large‐area, seasonal, multi‐temporal, multi‐spectral remote sensing approaches that are being applied to the reconstruction of the prehistoric hydrographical network of the Sutlej‐Yamuna interfluve, in northwest India (see later). Our remote sensing‐based method has been able to map more than 8000 km of relict water courses (Orengo and Petrie, 2017). During the course of that analysis, it became evident that the detection and mapping of topographic features such as levees, relict riverbeds, bluff lines and dune fields could not just increase the number of palaeorivers in our AOI, but also provide significant insights into their nature, behaviour and eventual disappearance. All these features are good examples of micro‐topographical features that extend over large areas and remain undetectable using current DSM/DTM visualization techniques.

## Method: Calculation of MSRM

MSRM shares a similar approach to that of pyramidal representations of large rasters used in computer graphics. Pyramids are usually created by smoothing the original raster with a low pass filter and then subsampling the smoothed image by a factor of two. The resulting raster follows the same process and the procedure is repeated to create an image stack for multiscale visualization. However, while pyramids are usually employed as a means of compression, MSRM uses the differences between pyramid levels to highlight features. MSRM's focus on topographic differences closely relates it to the local relief model (LRM) algorithm, which is a DSM‐based application of standard trend‐removal techniques that aimed to be applicable to the detection of micro‐topographies (Hesse, 2010). LRM is a technique for the filtering of high resolution DTMs that is based on the subtraction of a smoothed surface from the original DTM. This process results in a DEM in which only micro‐topographic features are visible. Hesse (2010) also included a step to make the visualization more natural by adding a filtered version of the original topography to the micro‐topographic model. LRM has become one of the most widespread and useful techniques for the analysis of micro‐topographies in archaeology (its original field of application) and beyond, and it is now included in standard geographic information system (GIS) packages, such as GRASS GIS. Although LRM was conceived for the filtering of DTMs derived from LiDAR data, it can potentially be applied to any type of DTM, as its basic unit of analysis is the raster cell, regardless of the actual size of the area it covers. However, LRM is restricted to the detection of the elements smaller than the kernel determined by the resolution of the DTM. MSRM aims to extend micro‐relief detection applications to multi‐scale features and by incorporating the use of multi‐scale DTMs, including those of global scope, to the interpretation of geomorphological features.

The generation of MSRMs consists of the development of several low pass filters of the original surface with different kernel sizes. The kernel diameters should ideally cover the sizes of all potential features to be detected. The results of consecutive low pass filters are subtracted between them, and each of the resulting relief models is summed. The summed model is then divided between the number of relief models in the following manner:

$${\text{MSMR}}^{x}=\frac{\sum_{i}^{n-1}\left(\mathrm{LPr}\left(i^{x}\right)-\mathrm{LPr}{\left(i+1\right)}^{x}\right)}{n-i}$$

Here LPr is the filtered surface with a low pass kernel radius (*r*) of *i* ^*x*^, for the first term of the summation, and (*i +* 1)^*x*^, for the second one; *x* is the exponent of a power function determining the scaling factor for the radius values, that is, the spacing of the kernel levels; *i*, is the lower bound of the summation index, *i* is also equivalent to the radii of the low pass filter in the first term of the summation when *x* = 1.

The kernel radius of the initial filtered surface *i* ^*x*^ should provide a resolution adequate to detect the minimum feature size. In cases in which no assumptions are made about the minimum size of the features to be detected *i* should equal zero. This would result in an initial surface LPr0^*x*^ in which the radius of the kernel equals zero), which corresponds to the original DSM. A value of zero for *i*, will extract the maximum of topographical information available in the DSM, but in cases where the minimum feature size to be detected is known, the following rule should be adopted in order to filter out unwanted features visible at the finest resolution and reduce the use of system resources:

$$i=\left\lfloor {\left(\left(f_{\min}-\mathrm{rr}\right)/2\mathrm{rr}\right)}^{\left(1/x\right)}\right\rfloor$$

where *f*_min_ is the minimum size of any feature to be detected, rr is the raster resolution and *x* the scaling factor employed. This equation can be divided in three components:

1. A calculation of the kernel radius *r* in pixels necessary to cover the minimum feature size. If kernel size in pixels equals 2*r* + rr and the minimum kernel size in pixels to cover *f*_min_ equals *f*_min_/rr, then the minimum kernel radius *r* equals (*f*_min_ − rr)/2rr. The resulting value equals the radius of the initial filtered surface, that is, the first term of the MSRM summation during its first iteration, if the exponential scaling factor *x* equals one. However, in order to allow scaling factors larger than one in the MSRM formula, the calculation of *i* requires the use of:
2. An exponent 1/*x* aimed to compensate the scaling factor *x*. The first term of the summation in the MSRM formula, LPr (*i* ^*x*^), includes the scaling factor in the calculation of the filter radius, which is necessary for the development of non‐linear spacing of the filtered surfaces. However, this initial filtered surface has to use a kernel size equal or smaller than *f*_min_. The use of this exponent ensures that the lower bound of the summation *i* compensates the scaling factor present in the first term of the MSRM summation.
3. The value resulting from the two previous operations is rounded to the lower integer (flooring operation indicated by the floor brackets in the formula earlier). This is to ensure that an integer number (since the radius is measured in pixels) will be employed with a smaller or equal kernel size than that of *f*_min_ for the development of the low pass filter.

Selecting an appropriate value for *n* should apply a similar formula to that described earlier:

$$n=\left\lceil {\left(\left(f_{\max}-\mathrm{rr}\right)/2\mathrm{rr}\right)}^{\left(1/x\right)}\right\rceil$$

where *f*_max_ is the maximum size of any feature to be detected. Note that *n* value resulting from this rule is rounded to the upper integer (ceiling operation indicated by the ceiling brackets in the formula earlier). This is to ensure that a low pass filter with an equal or larger kernel size than that of *f*_max_ will be employed. The *n* value is related to the number of surfaces to be generated: *n − i* equals the number of relief models (resulting from LPr(*i* ^*x*^) − LPr(*i* + 1)^*x*^) necessary for the generation of a MSRM covering both minimum and maximum feature sizes. Thus, *n −* 1 equals the upper bound of the summation. The subtraction of 1 to *n* is necessary as the second low pass radius of every term in the summation series adds 1 to the value of the first low pass radius and, therefore, the maximum kernel size is already included in the *n* − 1 relief model.

The scaling factor *x* is an important variable in this algorithm. It makes it possible to set the trade‐off between MSRM sensitivity and the range of feature sizes to be detected. The larger the scaling factor, the wider the range of feature sizes that will be included in the MSRM using an equal number of relief models. A higher scaling factor results in a lower number of surfaces employed for the calculation of the MSRM and, therefore, a significant reduction on the use of computing resources, which can be necessary given the large computing requirements of the MSRM algorithm. The number of relief models included in the operation and the range of values of the resulting MSRM keep an inverse proportional relation. In this regard, the use of a non‐linear spacing of the kernels levels (in which *x* > 1) provides a larger range of MSRM values than that of a linear spacing (*x* = 1), which results in an increase of the contrast of the image and slightly improves its visibility. However, a larger scaling factor (i.e. a larger spacing between kernel levels) would also imply a larger topographic difference between relief models, which could result in a loss of sensitivity of the MSRM to intermediate sized features. A more sensitive MSRM can be produced by keeping the increase of filter radii in consecutive filtered surfaces to a minimum: a scaling factor of 1 will produce an arithmetic (linear) scaling of the low pass filters’ radii. This would be convenient when there are no large differences between the sizes of features to be detected or their sizes are likely to be similar. Of course, the use of a linear scaling factor to cover a large range of feature sizes will imply a higher number of relief models and, consequently, a reduction in the range of values in the MSRM and a much higher use of computational resources.

When interpreting an MSRM, three characteristics of the resulting DEM need to be kept in mind: (1) MSRM does not provide a natural visualization – although the data represented in MSRM are based on real topography and they keep a proportional relationship with it, MSRM values are not a direct representation of relief or topographic differences, but an artificial way to visualize variable differences in height over large areas; (2) MSRM creates a topographic edge effect around features, which is not based on real topographic data, but is an artefact of the subtraction of filtered surfaces; and (3) it creates an edge effect surrounding the AOI, which equals the kernel size of the filter. This can easily be avoided by increasing the AOI accordingly, which should not constitute a problem when a global DSM is employed.

Another factor to take into account is the difference in elevation present in the AOI. When mountain and plain areas are analysed in conjunction the difference in the MSRM values in mountain areas can render the much smaller value range in the plain almost invisible when the data are displayed using a colour scale. The use of minimum and maximum values in accordance with the geomorphological character of each subzone is recommended in such cases. Given the use of multiple kernels sizes, MSRM tends to filter larger areas than other relief visualization tools. For this reason, the application of MSRM to mountainous or rugged terrain does not produce good results for the visualization of micro‐relief.

In order to improve its visualization, MSRM can be combined with digital illumination relief enhancing techniques, such as shaded relief models. These can help with the interpretation of the MSRM model by providing a more natural (if still unrealistic) visualization. However, before employing the MSRM surface to generate shaded relief models, it is necessary to transform the raster values. MSRM rasters include both positive (elevations) and negative values (depressions). Given the inability of shaded relief model algorithms implemented in most GIS software to deal with negative values, a raster with only positive values will need to be employed to generate the shaded relief map. This can be achieved easily by normalizing the MSRM raster values (a range of values of 0 to 255 is recommended) using standard raster calculator tools. The original un‐normalized MSRM surface can be colour scaled by standard deviations and made slightly transparent so that it can be combined with a shaded relief model of the normalized MSRM.

## Comparison with Other Common Relief Visualization Methods

**Figure 1:**
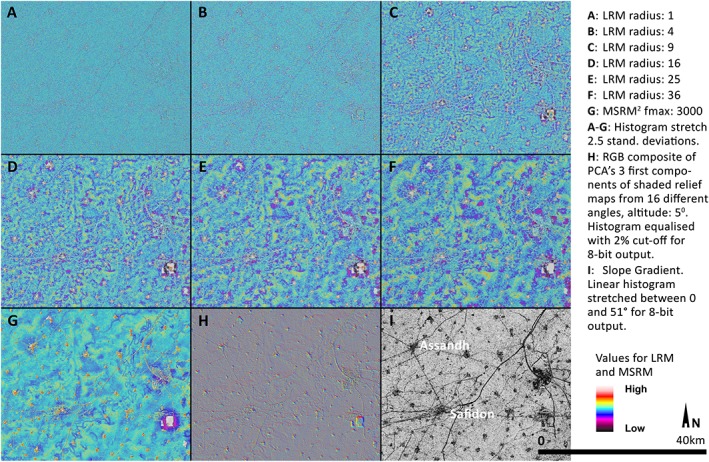
Comparison between multi‐scale relief model (MSRM) and other micro‐topography visualization methods. Local relief model (LRM) (A–F), MSRM (G), principal component analysis (PCA) of multi‐azimuth shaded relief maps (H) and Slope Gradient (I). LRM maps have been calculated using r.local. Relief module for GRASS 7.2 written by V. Petras and E. Goddard. PCA of multi‐azimuth shaded relief maps and Slope Gradient have been calculated using the Relief Visualization Toolbox 1.3 (Kokalj *et al*., 2016). [Colour figure can be viewed at http://wileyonlinelibrary.com]

In Figure 1, a comparison between different micro‐relief visualization methods including LRM, MSRM, PCA of multi‐azimuth shaded relief maps, and Slope Gradient is provided. In Figure 1 and those that follow, a colour scale formed by two diverging sequential palettes (warm tones for positive and cold tones for negative heights), topped and bottomed by white and black, respectively, has been created to highlight small differences in both positive and negative height. The use of any other diverging scale with enough contrast should provide satisfactory results.

The visualization methods selected in Figure 1 include DEM manipulation‐based (LRM, MSRM and Slope Gradient) and illumination‐based techniques (PCA of multi‐azimuth shaded relief maps) to provide a comparison between the two types of approach. The choice of PCA of multi‐view shaded relief maps and Slope Gradient aimed to include DEM visualization techniques that are not kernel‐size dependent. Figure 1 clearly shows the advantage of using a multi‐scalar approach to the detection of large micro‐features, in this case a series of palaeorivers in the region of Haryana, northwest India, with planforms ranging from sinuous to anastomosis. LRM is able to show many features using different kernel sizes. Roads and water channels appear clearly in images A to C depending on their size. Palaeorivers are most visible in images C to F. However, these images are unconnected, and their interpretation is difficult as they produce unnatural representations. Other kernel‐size dependent techniques such as Sky‐View Factor or Openness result in similar partial representations and features are visible according to how well they align with the kernel size employed. These other techniques are, therefore, inadequate for the detection of large natural micro‐features such as palaeorivers, which usually present varied sizes and topographic imprint along their route.

The MSRM image (Figure 1G) includes the same number of filtered surfaces using the same kernel radius as images A–F, but their integration is able to offer a more natural representation of the relief. All features (roads, channels, palaeorivers and large structures) are visible in a single image. The rivers are represented continuously and are interpretable within their specific topographic context.

Non‐kernel size‐dependent visualization techniques clearly highlight modern structures, but while Slope Gradient (Figure 1I) does not identify any natural feature, PCA of multi‐azimuth shaded relief maps (Figure 1H) is able to mark the subtle topographic depressions associated to palaeorivers. This is related to the very subtle nature of these features in which topographic change defining a feature expand through multiple cells leaving scarce relief to be identified by these techniques. MSRM multiple kernel size is able to overcome this problem by identifying topographic change at different scales and joining these to draw subtle topographic features over large areas.

## Application: Micro‐relief Analysis on a Continental Scale using Google Earth Engine© Code Editor, Repository and Cloud Computing Platform

The project entitled ‘Winter Rain, Summer Rain. Adaptation, Climate Change, Resilience and the Indus Civilisation’ or *TwoRains* is investigating the interplay and dynamics of winter and summer rainfall systems, the nature of human adaptation to the ecological conditions created by those systems, and the influence of climate change and water availability on the resilience of South Asia's Indus Civilization. A critical component of this overarching project is the reconstruction of the palaeoriver network in the study area of northwest India, the so‐called Sutlej‐Yamuna interfluve, which was a core area for this ancient civilization. The Sutlej‐Yamuna interfluve is a remarkably flat plain with a height difference of *c*. 100 m in over 300 km that is formed by quaternary alluvial deposits originating from the Himalayas (Srivastava *et al*., 2006). The area incorporates parts of the modern Indian states of Punjab, Haryana, and northern Rajasthan, and covers a total of more than 80 000 km^2^.

The form and visibility of palaeorivers in this area can be highly variable, depending upon the nature of the original river system, the amount of time since its abandonment, and the natural and human influenced landscape modification that has subsequently taken place. Although a palaeoriver in this region might be very wide it can also be characterized by relatively slight topographic depressions corresponding to the riverbed or elevation related to its levees or sedimentary accumulations along its course.

MSRM was conceived as an ideal tool to detect palaeorivers in this environment, map them in detail and define the topographic features associated to them and influencing their course, and also describing their character. For the analysis of the study area's topography three recently released medium‐resolution DSMs were employed: the TanDEM‐X©^DLR 2017^ (Wessel, 2016; Rizzoli *et al*., 2017) DSM, which is a 12 m/cell DSM developed by the German Aerospace Centre that was generated from bistatic X‐Band interferometric SAR acquisitions; and the USGS’ Shuttle Radar Topography Mission (SRTM) at 30 and 90 m/cell (Farr *et al*., 2007), which was also developed using interferometric SAR (see Table 1 for details of these DSMs).

**Table 1:** Digital surface model (DSM) sources employed and their characteristics

DSM	Spatial resolution	Absolute horizontal accuracy	Absolute vertical accuracy	Relative vertical accuracy	Coverage and release date
TanDEM‐X DEM	~12 m (0.4 arcsecond at equator)	< 10 m	< 10 m	2 m (slope ≤ 20%) 4 m (slope > 20%)	Global (97% of land mass) 2016
SRTM 30 m	~30 m (1 arcsecond at equator)	≤ 20 m	≤ 16 m	≤ 10 m	Global (80% of land mass) Late 2015
SRTM 90 m v.4	~90 m (3 arcseconds at equator)	≤ 20 m	≤ 16 m	≤ 10 m	Global (80% of land mass) 2008

Google Earth Engine© (GEE) (Google Earth Engine Team, 2015) was employed for the implementation and application of the MSRM algorithm. GEE is a web‐based geospatial computing platform with several inter‐related components. The Code Editor is an integrated development environment for GEE's JavaScript application programming interface. It allows users to write and run their own scripts using GEE's JavaScript implementation to import, process, analyse, visualize and export geospatial data. GEE also provides access to petabytes of satellite imagery, which includes SRTM 90 and 30 m/cell, but also many other continental and national‐scale, large, medium and high‐resolution DSMs. This functionality is very convenient as users can directly access DSMs and incorporate them in their scripts without the need to download and mosaic all scenes composing their study area. GEE also allows users to employ their own datasets, in our case the TanDEM‐X DSM, which is not publicly available and was made available to the *TwoRains* project by the German Aerospace Centre (DLR). Lastly, GEE allows users to run scripts through Google's cloud parallel computing infrastructure. MSRM can be extremely demanding on system computing resources. In the case of our research, this was particularly pressing as: (1) the study area extends over more than 80 000 km^2^; (2) the relatively high resolution of the TanDEM‐X DSM, which amounts to more than 2.5 GB of disk space; and (3) the large variability in size of the features of interest (from 500 m to more than 5000 m in length), which multiplies the number of surfaces to be generated.

The GEE JavaScript code generated for the reproduction of MSRM is provided as Supporting Information to this paper. The code has been prepared so that it is possible to apply the MSRM algorithm to any area and to enable the detection of any size of micro‐relief after it is pasted into GEE's Code Editor and executed (please, note that GEE requires registration, but it is otherwise free for non‐commercial purposes).

**Figure 2:**
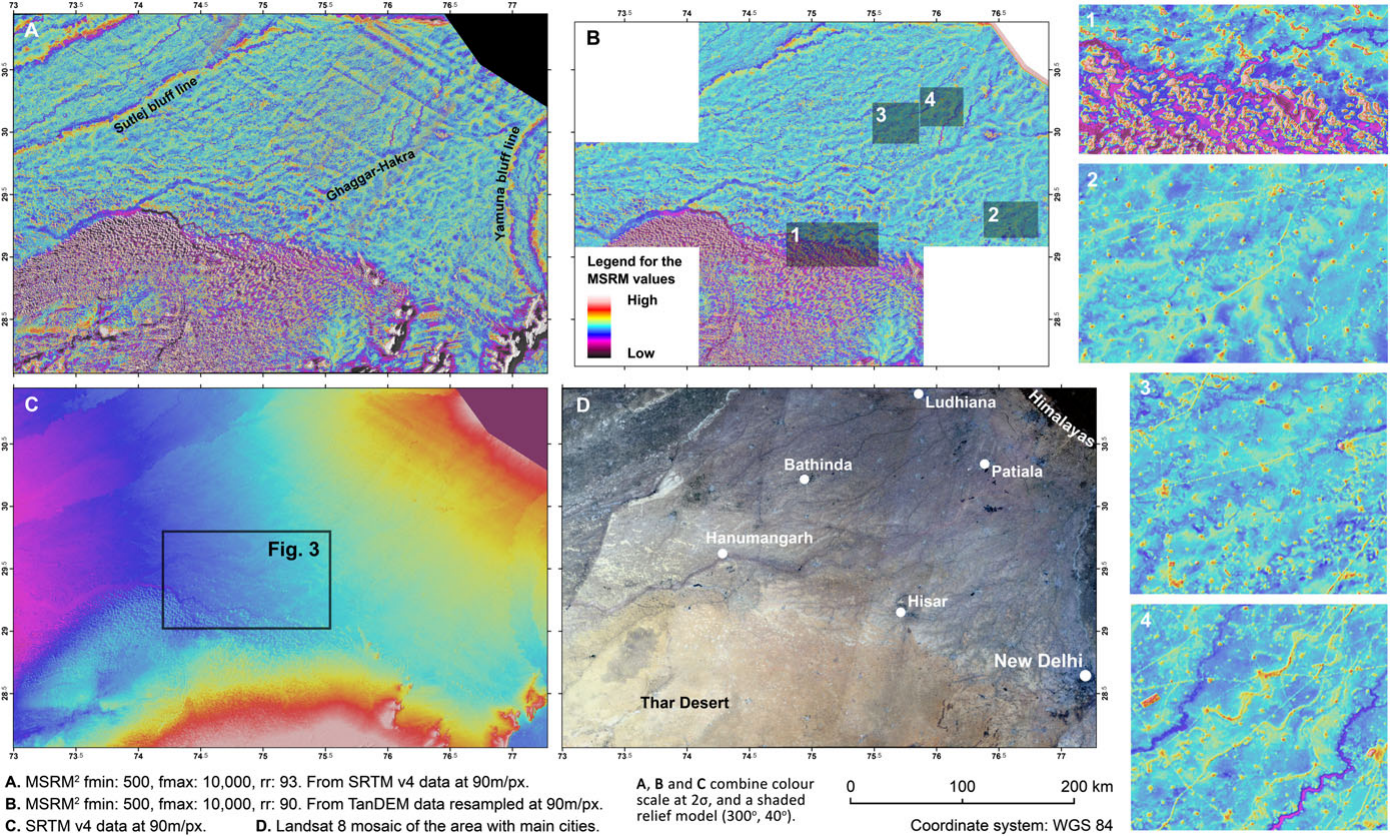
Application of the multi‐scale relief model (MSRM) algorithm over a very large area using different low‐resolution topographic sources. [Colour figure can be viewed at http://wileyonlinelibrary.com]

The application of MSRM to very large areas (Figure 2) can successfully identify a wide range of features of interest employing low‐resolution models. In Figure 2, MSRM (*x*: 2, *f*_min_: 500, *f*_max_: 10 000) is applied to both SRTM90 (Figure 2A) and TanDEM data resampled at 90 m/px for comparison purposes (Figure 2B, the availability of TanDEM data was restricted to this area). The resulting maps are able to identify a large number of micro‐reliefs of multiple sizes over a very large area. In Figures 2A and 2B, the Ghaggar‐Hakra palaeochannel, which reaches a width of 6 km in some parts of its route, can be identified crossing the study area diagonally from northeast to southwest. The mapping of the Ghaggar‐Hakra (often identified as the Vedic Sarasvati) and related hydrology, has been the focus of publications using multi‐spectral satellite imagery since the late 1970s (e.g. Ghose *et al*., 1979; Yashpal *et al*., 1980; Sharma *et al*., 1999; Gupta *et al*., 2004; Bhadra *et al*., 2009; Mehdi *et al*., 2016). A large number of relict rivers following this same general direction are also evident in the MSRM maps. These palaeorivers, some of them tributaries of the Ghaggar‐Hakra are, for the most part, invisible in topographic data (Figure 2C) and satellite imagery (Figure 2D). Another large palaeochannel running close to Hisar and joining the Ghaggar‐Hakra below Hanumangarh can also be identified in the MSRM maps (Figure 2.1). This relict watercourse marks the edge of the northern dune fields of the Thar desert. MSRM analysis makes it possible to clearly distinguish the morphology of these dunes, to identify several isolated dune fronts to the north of this palaeoriver – many of which are not visible in other sources, and to analyse the interplay between dunes and palaeoriver. MSRM has also allowed the study of the morphology of these palaeorivers, as it is able to detect successive meandering and anastomosing river courses in great detail even using low‐resolution DSMs (Figures 2.2 and 2.3). River levees, when existent, were also clearly reflected (Figure 2.4).

Most global or quasi‐global DSMs such as ALOS, ASTER, GDEM and SRTM present inconsistencies, noise, data holes and stripping: for example, diagonal stripes at different scales that are related to the orbital path of the space shuttle can be particularly pronounced in flat terrain. Although SRTM is usually considered of higher quality than that of the other freely available global DSMs, stripping in several directions and at different scales is still fairly common in SRTM data. Although stripping significantly hinders the interpretation of micro‐topographies, many of the features visible in TanDEM data, which do not show any stripping, can also be found using freely available SRTM data.

**Figure 3:**
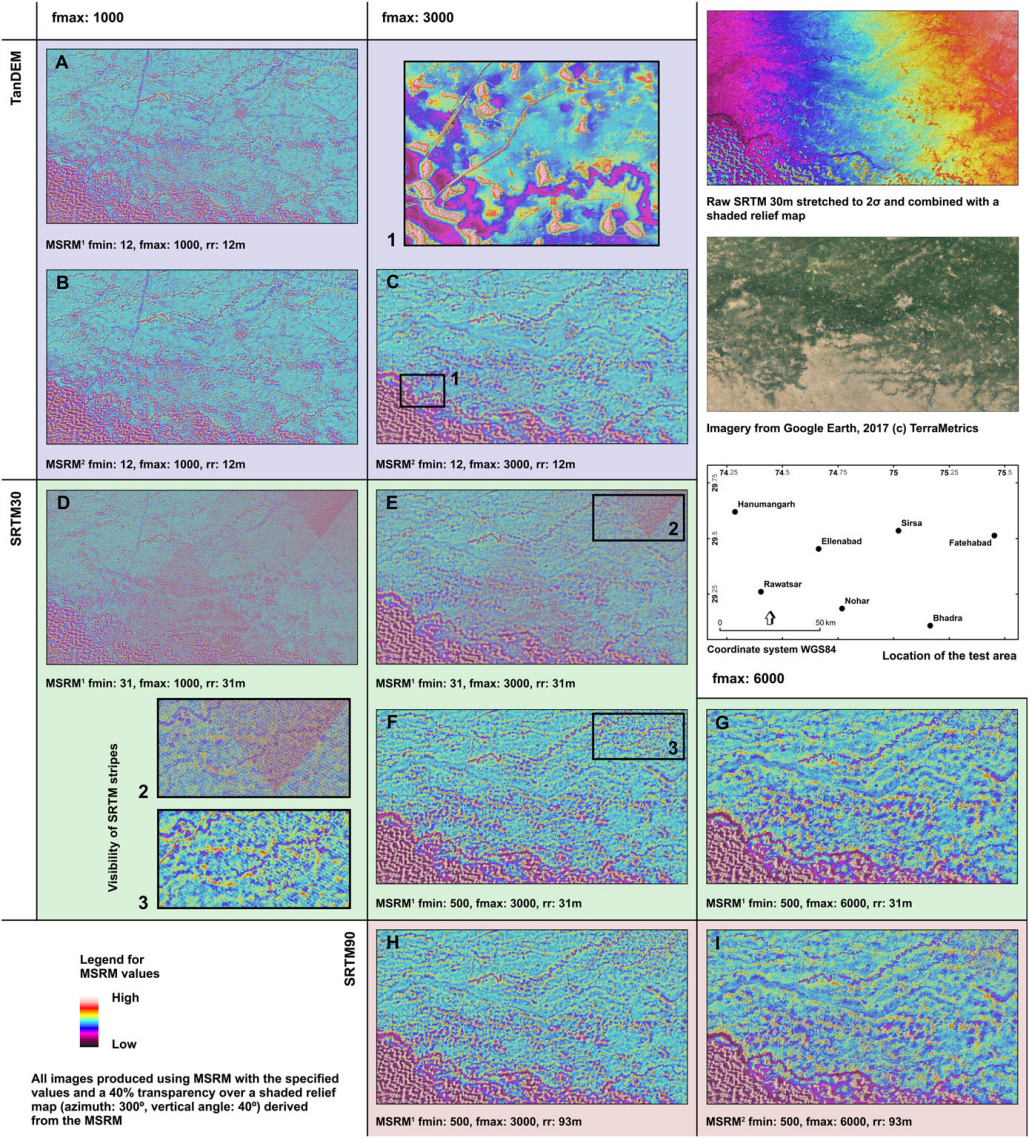
Comparison of multi‐scale relief model (MSRM) results using different parameters and topographic sources. [Colour figure can be viewed at http://wileyonlinelibrary.com]

A comparison of the application of MSRM using different parameters and DSMs in a relatively small area with a high variability of feature types is provided in Figure 3. The MSRM‐derived micro‐relief maps are also compared with the original DSM and a natural colour composite derived from medium resolution satellite imagery. Figure 3 demonstrates the behaviour and best application of MSRM. It clearly shows the importance of selecting adequate values for the higher and the lower cutoff frequency of the filters: *f*_max_ and *f*_min_. The *f*_max_ values, as expected, dictate the maximum feature size to be detected. Only minor palaeorivers are shown using a *f*_max_ value of 1000, the large palaeoriver to the south of the Ghaggar‐Hakra (Figure 2.1) can only be detected using a *f*_max_ value of 3000 and the Ghaggar‐Hakra riverbed becomes clear using *f*_max_ values above 5000. However, the bluff lines of the Ghaggar‐Hakra, being a topographic feature themselves, are slightly visible with a *f*_max_ value of 1000 and clearly delineated at *f*_max_ 3000. These are all visible using a *f*_max_ value of 10 000 (Figures 2A and 2B), which illustrates the convenience of using a large filter radius for exploratory topographic analysis as a large *f*_max_ value will also include all smaller features of interest.

**Figure 4:**
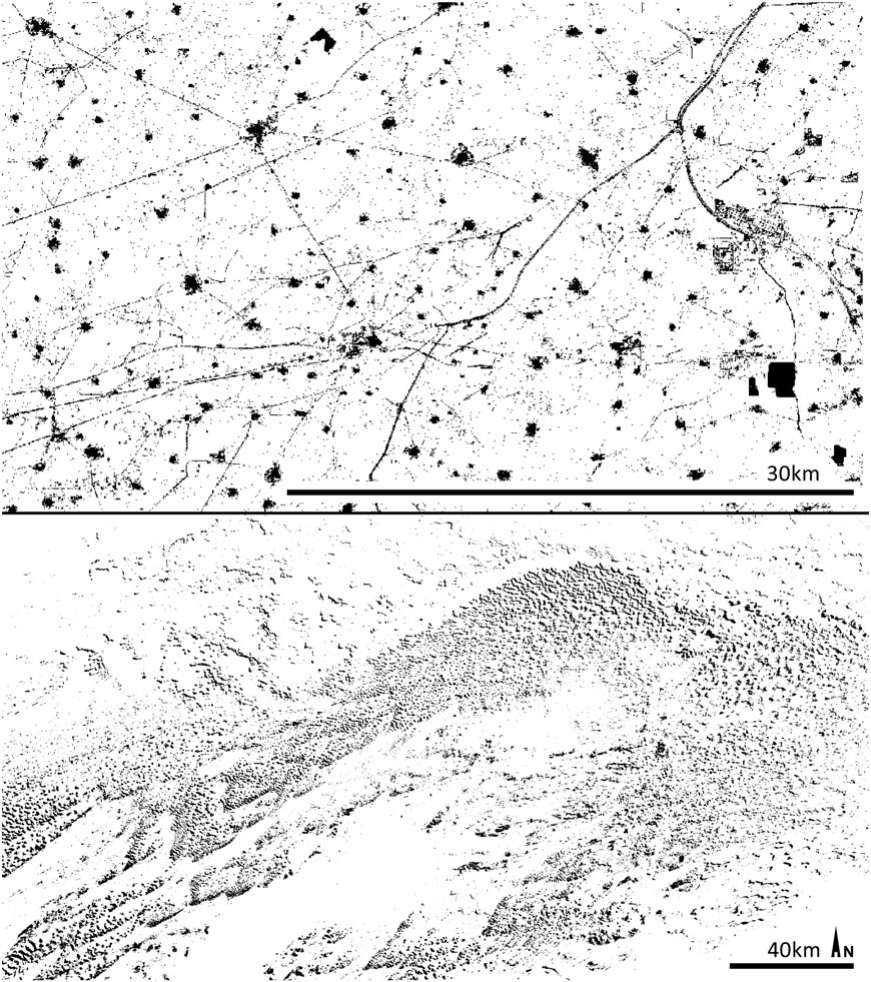
Examples of extraction of features using multi‐scale relief model's (MSRM's) *f*_min_ and *f*_max_ value ranges and reclassification of MSRM data. The upper image shows the same area than that at Figure 1. All sharp prominent features including roads, water channels, towns and industrial complexes have been isolated. The lower image represents the north‐eastern sector of the Thar desert, were dunes have been identified through their relative height range in MSRM.

Low values for *f*_min_ in Figures 3A, 3B and 3D (where *f*_min_ is set to its minimum value, which equals the original DSM resolution) have resulted in the inclusion of many features of no geomorphological interest, the most visible of those are probably the large water channels criss‐crossing the area. In Figures 3D and 3E, which make use of SRTM30 data, the visibility of smaller stripes is boosted by the application of MSRM, clearly hindering the detection of other features. An adoption of a *f*_min_ value above the size of these stripes and water channels can filter these out and increase the visibility of palaeorivers and other geomorphological features when using SRTM data (compare with Figures 3E and 3F). In relation to this, the combination of kernels of different size in MSRM creates a topographic baseline that can be used to isolate features with a constant elevation above that baseline such as channels or towns. These would have presented different height values in a DSM depending on the elevation of the area in which they are measured. MSRM, however, makes evident their constant relative height. This can be used to extract features (using a slice or reclassification method) according to their relative prominence (see several examples in Figure 4). The extracted features can then be used for other purposes such as DSM filtering (e.g. using prominent sliced features to crop the DSM and then applying a filling algorithm), as part of automatic classification methods and so on.

The use of a scaling factor of two in Figures 3B, 3C and 3I, does not significantly reduce the visibility of features, providing very similar results to the use of a scaling factor of one but with a significantly reduced processing time and a higher contrast as a result of the larger range of values that the use of a lower number of relief surfaces implies. However, a scaling factor of one is recommended when the range size of features to be detected is small and a scaling factor of two would result in the generation of very few relief models.

The application of MSRM in the Sutlej‐Yamuna interfluve clearly shows this algorithm's capacity to detect otherwise invisible features and to extract micro‐relief information from all resolutions available. The only limitations are the DSM's spatial resolution, which equals the minimum feature size to be detected, and the DSM's quality. In any case, large‐scale micro‐topographical and medium‐size features such as palaeorivers and associated levees and the morphology of dune fields can be clearly appreciated in each instance. This capability is of exceptional relevance for the study of the plains of northwest India, as many of these features are not visible employing high‐resolution satellite imagery nor unfiltered or colour‐scaled DSMs, which are the most common sources for geomorphological analysis as of today.

The application of MSRM in the study area has allowed us to map more than 10 000 km of palaeorivers, though their analysis in terms of cultural and environmental significance lies beyond the scope of this paper and will be the focus of future publications. Although many of these palaeochannels had already been detected using seasonal, multi‐temporal, multi‐spectral imagery analysis, many others are only reflected as subtle micro‐reliefs of large size. In this regard, MSRM cannot be regarded as a complete substitute for multispectral data analysis (as some palaeorivers did not leave any topographic indication of their former course), but as an important complement to these methods. MSRM, however, provides the majority of the data on palaeoriver courses in the study area and has enabled us to complete the ancient palaeoriver network derived from multi‐spectral imagery analysis (Orengo and Petrie, 2017). More importantly, MSRM has provided essential complementary information, such as the size of the rivers’ channels and the presence and morphology of levees, and it has been able to detect previously unknown bluff lines created by relict large rivers. In the course of the MSRM‐based palaeo‐hydrological reconstruction, the usefulness of MSRM for the detailed mapping of other features that are defined by large micro‐topographies, such as dune fields and their relation to relict riverbeds, has also become evident.

## Conclusions

MSRM offers a way of extracting multi‐scale topographic information available within high‐, medium‐ and low‐resolution DSMs that would remain invisible using current DSM visualization techniques. In this regard, MSRM can provide a new important tool in the geomorphologist's toolbox, overcoming the current dependency on the availability of good quality (in terms of resolution, visibility and environmental conditions) aerial and satellite imagery and providing images, which are directly related to the object of study: the topography and shape of the feature of interest. The application of MSRM can also be beneficial to all other research fields aiming to interpret small terrain differences, such as engineering, landscape architecture, archaeology and so on. MSRM can also be useful in evaluating the quality of topographic data in particular regarding the identification of stripes caused during the data acquisition process. A judicious selection of MSRM parameters can provide a simple way of masking these elements, improving the DSM capacity to reflect topographic change. MSRM's capacity to map features of constant relative height can also be used to identify and extract these for mapping purposes or to be implemented as part of other analyses. We provide the code employed to implement the algorithm in GEE as Supporting Information with the hope that other researchers will be able to use it in accordance with their own interests, evaluate it and improve it. In this regard, the use of GEE offers a way to make this computing‐intensive algorithm accessible to researchers lacking access to high performance computing resources, GIS software and or programming/GIS skills.

## Supporting information

Data S1. Supporting Information.
